# Genetically evaluating the causal role of peripheral immune cells in colorectal cancer: a two-sample Mendelian randomization study

**DOI:** 10.1186/s12885-024-12515-z

**Published:** 2024-06-21

**Authors:** Runze Huang, Xin Jin, Ziting Jiang, Yixiu Wang, Yibin Wu, Lu Wang, Weiping Zhu

**Affiliations:** 1 Department of Hepatic Surgery, Fudan University Shanghai Cancer Center, Fudan University, Shanghai, 200032 People’s Republic of China; 2 Department of Oncology, Shanghai Medical College, Fudan University, Shanghai, China

**Keywords:** Colorectal cancer, Mendelian randomization, Peripheral blood immune cell, Therapeutic target

## Abstract

**Background:**

Investigating novel therapeutic strategies for colorectal cancer (CRC) is imperative. However, there is limited research on the use of drugs to target peripheral blood immune cells in this context. To address this gap, we performed a two-sample Mendelian randomization (MR) analysis to identify potential therapeutic targets for CRC.

**Methods:**

We applied two-sample MR to identify the causal relationship between peripheral blood immune cells and CRC. GWAS data were obtained from the IEU OPEN GWAS project. Based on the implications from the MR results, we conducted a comprehensive database search and genetic analysis to explore potential underlying mechanisms. We predicted miRNAs for each gene and employed extensive research for potential therapeutic applications.

**Results:**

We have identified causal associations between two peripheral immune cells and colorectal cancer. Activated & resting Treg %CD4 + cell was positively associated with the risks of CRC, while DN (CD4-CD8-) %leukocyte cell exhibited a protective role in tumor progression. NEK7 (NIMA related kinase 7) and LHX9 (LIM homeobox 9) expressed in Treg cells were positively associated with CRC risks and may play a vital role in carcinogenesis.

**Conclusions:**

This study identified causal relationship between peripheral immune cell and CRC. Treg and DN T cells were implicated to own promoting and inhibiting effects on CRC progression respectively. NEK7 and LHX9 in Treg cells were identified as potential biotarget for antitumor therapies.

**Supplementary Information:**

The online version contains supplementary material available at 10.1186/s12885-024-12515-z.

## Introduction

Colorectal cancer (CRC) is among the most prevalent solid tumors in the digestive system, resulting in the death of hundreds of thousands of individuals each year. Despite the promising results shown in many recent studies on CRC treatment, curing this common and highly prevalent cancer remains a formidable challenge. Nevertheless, an increasing body of evidence indicates that the identification of mutated genes through molecular profiling and subsequent targeted therapies represent a highly promising avenue in the field. By targeting cancer cells or immune cells within tumor tissues, this precision strategy is demonstrating potent therapeutic efficacy and significant advantages. However, the significance and therapeutic potential of this approach are not limited solely to immune cells within the tissue. Many studies have indicated that immune cells present in the circulating blood also hold significant importance, serving as promising sources of potential biomarkers [1] and playing a role in influencing therapeutic outcomes [2] and various other aspects. In the realm of colorectal cancer therapy, this tendency continues to rise unabated [3, 4].

T regulatory (Treg) cells, an indispensable component of the normal immune system involved in maintaining immune homeostasis, play a pivotal role in preventing autoimmune diseases. However, they are often found to be associated with a poor clinical prognosis in cancer patients, and can also be accumulated to impairing anti-tumor immunity and even directly promoting tumor progression in certain types of cancer [5, 6]. Previous studies have observed a high abundance of Treg cells in the peripheral blood in patient with gastrointestinal tumors [7], leading to significantly stronger immune suppressive activity [8]. As a cell population characterized by a high degree of heterogeneity, novel molecular signatures, and distinct functional attributes [9], peripheral Treg cells may represent a significant source of the Treg cells within tumor tissues. Meanwhile, impeding this recruitment could inhibit the proliferation of cancer cells [10]. Additionally, circulating Treg cells are closely linked with more malignant histopathological conditions [11, 12] and elevated tumor markers [13], suggesting that they may exhibit pro-tumorigenic tendencies and functions in certain contexts. Therefore, it is essential to explore whether circulating Treg cells also possess a causal relationship with CRC and to identify potential mechanism and available therapeutic targets.

Similar to Treg cells, CD3 + CD4-CD8- double negative (DN) T cells constitute another crucial subgroup within T cells, playing an essential role in immune homeostasis. Although they are rarely found in the peripheral blood, in vitro and preclinical studies have confirmed their robust capacity to prevent tumorigenesis [14] and demonstrate cytotoxicity against cancer cells [15]. Studies have observed a reduction in the levels of circulating DN cells in cancer patients, which is closely correlated with tumor burden [16]. This trend is also significantly present in CRC, suggesting that it may play an important role [17]. However, studies on the causal relationship between peripheral DN cells and CRC are few and far between, with more research focused on their therapeutic applications, as well as on other tumors or non-tumor diseases. In this context, it is essential to investigate whether there is a causal relationship between these two subtypes of T cells in the peripheral circulation and colorectal cancer. If key targets within these cells can be treated with drugs, it would also be of great significance.

Mendelian randomization (MR) is a statistical technique that relies on the principles of Mendelian independent distribution law. By utilizing genetic variants as instrumental variables, MR strengthens causal inference in relation to specific exposures. One of the key advantages of MR is its reduced susceptibility to confounding biases, as genetic variants are randomly assigned at conception, minimizing associations with environmental and self-adopted factors. Previous investigations have revealed significant correlations between Treg cells, DN cells and colorectal cancer [17, 18]. Moreover, a recent genome-wide association study (GWAS) has identified more than 700 immune cell traits in human peripheral blood. This groundbreaking discovery presents great opportunities to delve into the intricate realms of peripheral immunity and its implications in diverse pathological contexts [19]. To broaden our understanding in the field of CRC treatment, we perform a two-sample MR analysis on colorectal cancer to explore potential therapeutic strategy in the medication industry.

## Materials and methods

### Study design and ethics permit

**Fig. 1:**
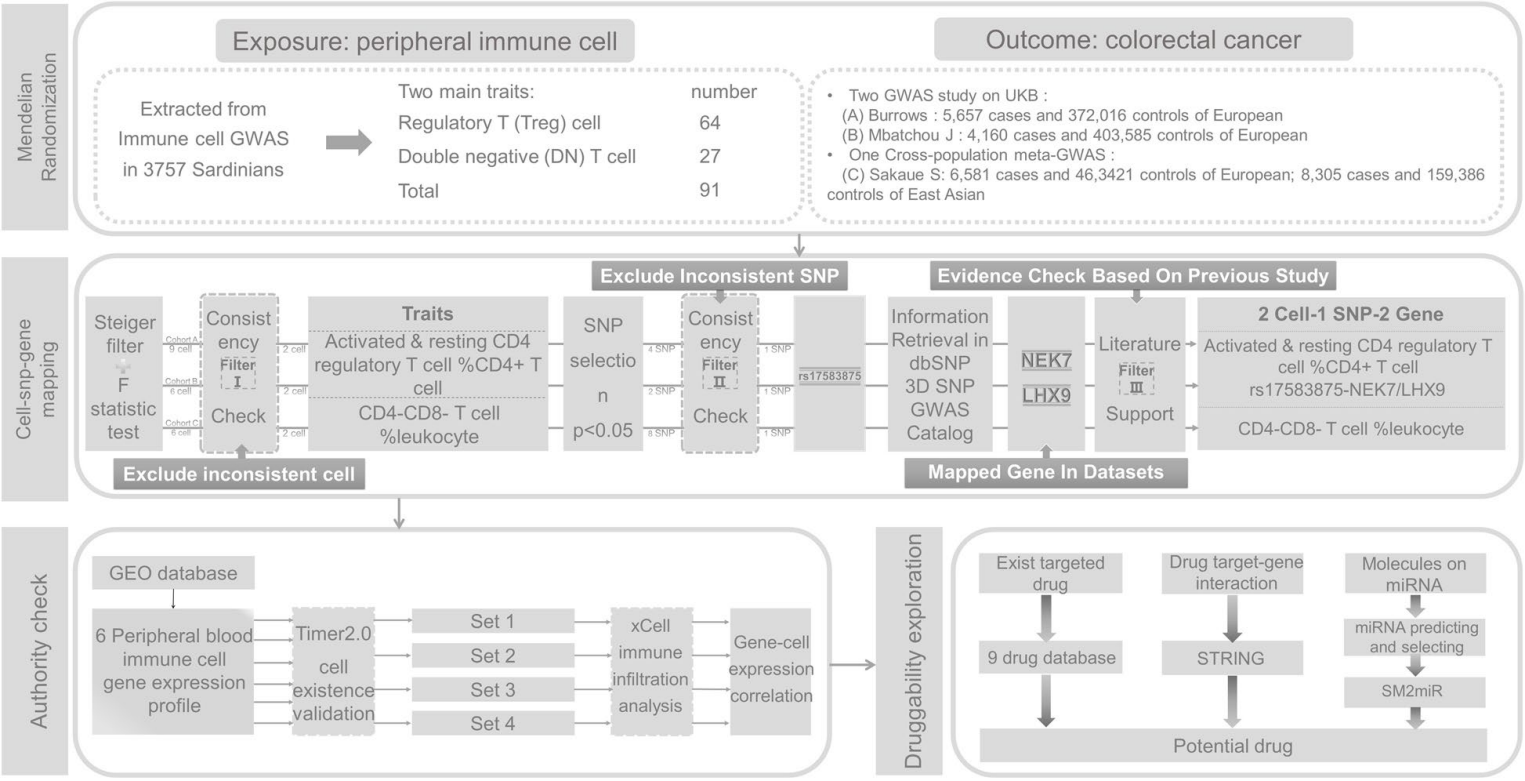
Study design. NEK7, NIMA related kinase 7; LHX9, LIM homeobox 9; GWAS, genome-wide association study

The overall study design is presented in Fig. 1. The study was based on publicly available data from large-scale genome-wide association study on immune cell and colorectal cancer. Included studies had been approved by corresponding ethical review committees.

### Immune cell data sources

Immune traits statistics of Treg cells and DN cells were extracted from a large-scale genome-wide association study [19], which are publicly available from the GWAS Catalog (https://www.ebi.ac.uk/gwas/). After filtering for the cell of interest, 91 traits were saved for further study, including absolute cell (AC) counts (*n* = 17), median fluorescence intensities (MFI) counts (*n* = 40) and relative cell (RC) counts (*n* = 34). These traits include Treg, Maturation stages of T cell and TBNK panel and relevant information are presented in Supplementary Table S2. It is worth noting that we only included Treg cells as defined by conventional criteria, thereby excluding other cell populations that may exhibit high expression of CD25 or other characteristics but are not classified as Treg cells. Furthermore, the dataset does not include double-negative B cells, hence a more detailed analysis of this subtype was not included. The original study was based on a population cohort of 3,757 Sardinians that tests approximately 20 million SNPs in 731 immunophenotypes genotyped with high-density arrays or specific sequence-based reference panel.

### Genome-wide association study (GWAS) data sources for CRC

We extracted genome-wide association study(GWAS) summary data for CRC from two different study on the UK Biobank [20, 21] and a cross-population analysis [22] through the *IEU Open GWAS* project (https://gwas.mrcieu.ac.uk/). Detailed information was documented in Supplementary Table S1. The UK Biobank is a population-based multicenter cohort study consisting of approximately 500,000 participants between ages 40 and 70. One study performed GWAS for pan-cancer and site-specific cancers identified by linkage to the UK CancerRegistry (updated to April 2019). After association statistics, a total number of 377,673 European individuals (Ncase = 5,657, Ncontrol = 372,016) were involved. Another UKB study introduced a novel and efficient machine-learning method named REGENIE and demonstrated the accuracy on 407,746 individuals (case–control ratio = 1:97). The cross-population summary data published in 2021 were performed on 470,002 European (Ncase = 6,581, Ncontrol = 463,421) and 167,691 East Asian (Ncase = 8,305, Ncontrol = 159,386) individuals with approximately 24 million variants detected.

### Mendelian randomization analysis

Mendelian randomization is an epidemiological approach that employs genetic variants as instrumental variables to infer causality between exposures and outcomes. This method relies on the principle that alleles are randomly assorted during gamete formation, mirroring a randomized trial's structure. It assumes that the genetic variants are associated with the exposure of interest, affect the outcome only through the exposure, and are not confounded by other factors. We performed two-sample MR analysis to capture the causal association between immune cells and colorectal cancer based on TwoSampleMR R package (github.com/MRCIEU/TwoSampleMR, version 0.5.7). MR analyses were conducted on each dataset acquired for CRC, and the results were recorded in Supplementary Table S3 ~ S6. We employed inverse variance weighting (IVW) as the primary approach and supplemented with other robust tests, including MR-Egger, weighted median and weighted mode methods, which rely on different assumptions than IVW. To avoid the effect of horizontal pleiotropy, MR-egger, leave-one-out and MR-PRESSO were adopted while Cochran’s Q statistic and *p* values were used to test the heterogeneity of all eligible instrumental variables (IVs). After excluding all SNPs with potential pleiotropy indicated by MR-PRESSO, we conducted a comprehensive analysis to ensure that the results were not influenced by pleiotropy. In accordance with recent studies, a significance level of 1 × 10^-5 was applied to the instrumental variables for each immune trait, along with a stringent clumping threshold (linkage disequilibrium [LD] r^2 threshold < 0.001 within 10,000 kb distance). For CRC, the selected SNPs were required to have a significance level below 5 × 10^-8. We employed Steiger's test and calculated the F-statistics to ensure all SNPs possess strong efficacy. Additionally, we utilized forest plots and scatter plots to assess and visualize the correlation of SNPs (Supplementary Figure S1-S2).

### SNP mapping and analysis

We employed several database to obtain relevant information for candidate SNPs, including the Functional Mapping and Annotation (FUMA) [23] and GWAS Catalog. By incorporating a multitude of biological resources and integrating associated data, FUMA enhances the richness and significance of results for GWAS studies. We conducted our analysis using the default settings of FUMA's SNP2GENE module, such as employing the 1000 Genomes Project Phase 3 reference panel for LD calculations with a threshold of 250 kb for LD blocks.

The GWAS Catalog contains a vast array of data from genome-wide association studies, including the localization information for many SNPs. The functionality of these SNPs has been thoroughly validated by other research, lending them greater credibility. Therefore, we retained the genes identified by both FUMA and the GWAS Catalog for subsequent analysis.

We also conducted an extensive document retrieval on the identified gene to verify and explore a potential role in tumorigenesis. Peripheral blood gene expression data from CRC patients and healthy individuals were obtained from the Gene Expression Omnibus (GEO) database (https://www.ncbi.nlm.nih.gov/geo/), and relevant information were documented in Supplementary Table S1. We also utilized GEO2R for differential gene expression analysis and performed visualization in R.

xCell is a robust gene signature method that has capability to identify up to 64 immune and stromal cell types in humans across various data sources. The correlation between immune cell types and gene expression levels was confirmed by combining xCell analysis with gene expression profiles, and the results were visualized in figures. xCell [24] analysis was conducted using the xCell R package (v1.2.0), and all relevant R code is publicly available on GitHub.

### miRNA targeting

The miRNet platform is a comprehensive network visualization tool that integrates 14 different databases, including TarBase, miRTarBase, miRecords, miRanda, miR2Disease, HMDD, PhenomiR, SM2miR, PharmacomiR, EpimiR, starBase, TransmiR, ADmiRE, and TAM 2.0. We use the miRNet database (https://www.mirnet.ca/) to identify corresponding miRNAs in peripheral blood and visualized the result in a circular chart. To assess their clinical relevance and expression in blood, we referred to the OncomiR [25] and dbDEMC database [26]. In the OncomiR database, we set the significance threshold at 0.05 for each section. The analysis was performed on both the tumor stage & grade part and the expression part, including data from both COAD and READ. In the dbDEMC database, the focus was on examining the miRNA expression profile in blood. To be considered qualified, the miRNAs had to meet specific criteria, including experimental validation of their close functional relationship with target genes, high expression levels in peripheral blood, and association with at least one clinical trait.

### Drug discovery

We conducted an extensive search across a range of prestigious databases, including the Connectivity Map, DrugBank, MedChemExpress (MCE), depmap, GeneCards, PharmGKB, the Drug-Gene Interaction database (DGIdb), The Therapeutic Target Database (TTD), the ChEMBL, and the PatSnap database. Additionally, we utilized the SM2miR database to obtain comprehensive drug information specifically related to the identified miRNAs. To further explore the draggability, we analyzed the interactions between commonly used clinical drugs and their target genes at the protein level. This investigation was carried out using the highly regarded STRING database (https://cn.string-db.org/). All potential effective small molecules were documented and classified, with their names, details and regulatory approval status up to date.

## Results

### Association between immunophenotypes and colorectal cancer

We extracted GWAS data related to Treg cells and DN cells traits from previous research, encompassing a total of 91 immune phenotypes. Following the two-sample MR analysis on three different outcome data, there are 9, 6, 6 cell types in cohort A, B, C that show significant correlation with CRC (*p* < 0.05). We initially conducted a preliminary screening of these cells for consistency check, excluding cells that did not show significant association across all of these cohorts. The results indicated that both Activated & resting CD4 regulatory T cell %CD4 T cell (Treg panel) and DN (CD4-CD8-) T cell % leukocyte (TBNK panel) demonstrated significant causal associations with colorectal cancer across various research cohorts. Based on the IVW method, Activated & resting Treg %CD4 + cells show a positive correlation with risk of colorectal cancer, while DN (CD4-CD8-) %leukocyte cells is suggested to have a protective effect against CRC **(**Table 1**)**. The Steiger test and the outcomes of other four approaches further corroborate the conclusion stated above, and the F statistics of all variants were all > 10. Collectively, these results suggest that certain subtypes of Treg cells and DN cells do possess functional aspects that either promote or inhibit tumor development, but the specific underlying factors remain unclear.

**Table 1:** Main results for Mendelian randomization

Traits	Method	Nsnp	BETA	SE	*P* value	OR	OR_lci95	OR_uci95
**UKB Group A**								
** Activated & resting Treg %CD4 + **	MR Egger	26	0.0017	0.0004	0.0002	1.0018	1.001	1.0026
	Weighted median		0.0016	0.0005	0.0002	1.0017	1.001	1.0026
	Inverse variance weighted		0.0011	0.0003	0.0004	1.0011	1.0004	1.0016
	Weighted mode		0.0017	0.0005	0.005	1.0017	1.001	1.0028
** DN (CD4-CD8-) %leukocyte**	MR Egger	18	-0.0009	0.0004	0.049	0.9991	0.9983	0.9999
	Weighted median		-0.001	0.0005	0.0393	0.9990	0.9981	0.9999
	Inverse variance weighted		-0.001	0.0003	0.00197	0.9990	0.9984	0.9997
	Weighted mode		-0.001	0.0004	0.02002	0.9990	0.9983	0.9998
**UKB Group B**								
** Activated & resting Treg %CD4 + **	MR Egger	26	0.0953	0.0303	0.004	1.10	1.0367	1.1672
	Weighted median		0.1119	0.0325	0.0006	1.1184	1.0494	1.1920
	Inverse variance weighted		0.0609	0.0213	0.0043	1.0628	1.0193	1.1082
	Weighted mode		0.1057	0.0347	0.0054	1.1115	1.0384	1.1897
** DN (CD4-CD8-) %leukocyte**	MR Egger	19	-0.0386	0.0299	0.2152	0.9622	0.9073	1.0203
	Weighted median		-0.0395	0.0343	0.2492	0.9613	0.8988	1.0281
	Inverse variance weighted		-0.0462	0.0235	0.0495	0.9548	0.9118	0.9999
	Weighted mode		-0.0527	0.0287	0.1992	0.9624	0.9097	1.0182
**Meta Group C**								
** Activated & resting Treg %CD4 + **	MR Egger	29	0.0615	0.0273	0.0330	1.0634	1.0079	1.1219
	Weighted median		0.0173	0.0244	0.4794	1.0174	0.9698	1.0674
	Inverse variance weighted		0.0536	0.0185	0.0038	1.0551	1.0175	1.0940
	Weighted mode		0.0189	0.0281	0.5071	1.0190	0.9645	1.0766
** DN (CD4-CD8-) %leukocyte**	MR Egger	20	-0.0917	0.0260	0.0024	0.9124	0.8671	0.9600
	Weighted median		-0.0730	0.0258	0.0046	0.9296	0.8838	0.9778
	Inverse variance weighted		-0.0539	0.0211	0.0106	0.9475	0.9092	0.9875
	Weighted mode		-0.0674	0.0235	0.0100	0.9348	0.8927	0.9790

*OR* Odds ratio, *DN* Double negative, *Nsnp* Number of SNPs

**Fig. 2:**
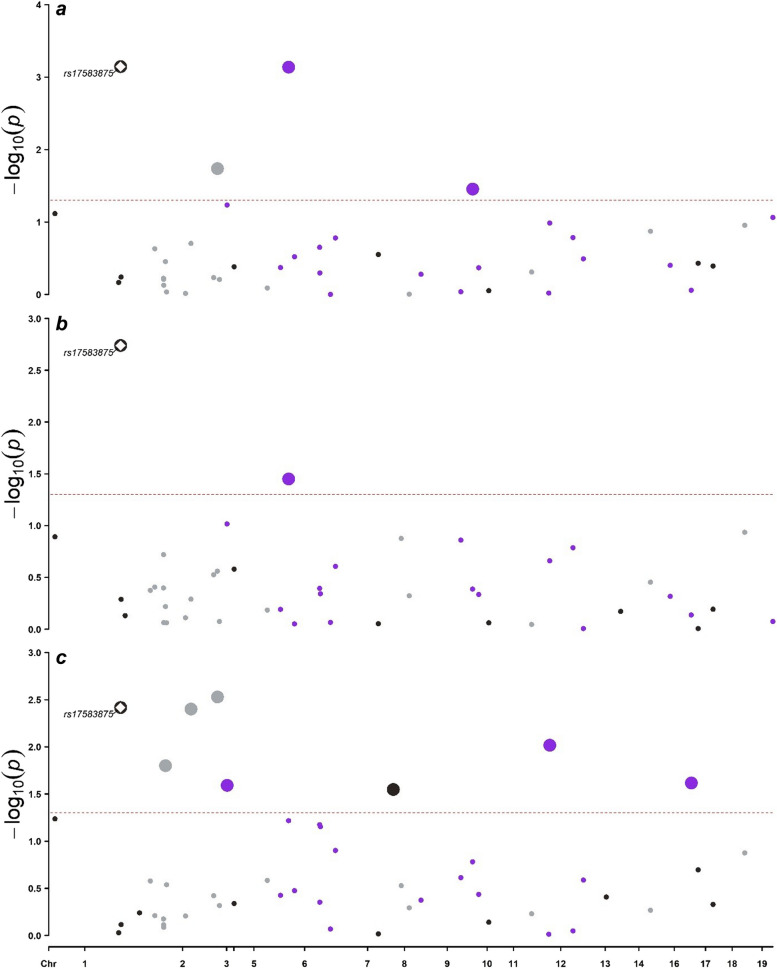
Manhattan plots for associations of SNPs within identified immune cells with colorectal cancer in MR analysis. **a** Associations of SNPs within identified Treg cells and DN T cells subgroup with CRC in UKB A group (Burrows’s). **b** Associations of SNPs within identified Treg cells and DN T cells subgroup with CRC in UKB B group (Mbatchou J’s). **c** Associations of SNPs within identified Treg cells and DN T cells subgroup with CRC in meta-c group (Sakaue S ’s). *P* value for significant threshold is set at a level of 0.05 (two-sample MR analysis). Labelled SNP shows close association across all sample sets. Results are plotted by chromosome position

To gain a deeper understanding of the potential influences of these cells, it is essential to conduct additional studies on gene level using instrumental variants. Thus, we identified a set of primary candidate single nucleotide polymorphisms (SNPs) that showing significant associations (*p* < 0.05) with the outcome. Similarly, there are 4, 2, 8 SNPs presences significancy in the mentioned group respectively. To ensure the robustness and consistency of the results, we conducted the second screening to identify variants with enhanced efficacy. In Activated & resting Treg %CD4 + cells, rs17583875 exhibits strong causal relationship across cohorts while no shared SNP was found in DN (CD4-CD8-) %leukocyte (Fig. 2). We additionally utilized the Wald ratio method to analyze the selected SNP, and observed that the odds ratio value exhibited a consonant trend with its cell type. Overall, rs17583875 Activated & resting CD4 regulatory T cell %CD4 T cell and DN (CD4-CD8-) T cell % leukocyte have been confirmed as qualified factors. Additionally, for a clearer and more comprehensive presentation of the MR results, we also utilized visual analysis tools, such as scatter plots and forest plots (Supplementary Figure S1- S2).

We also employed several methods for sensitivity analysis. After removing the outlier SNP identified by MR-PRESSO, the comprehensive analysis revealed no significant presence of pleiotropy or heterogeneity that would impact the overall results.

### SNP mapping and gene validation

**Fig. 3:**
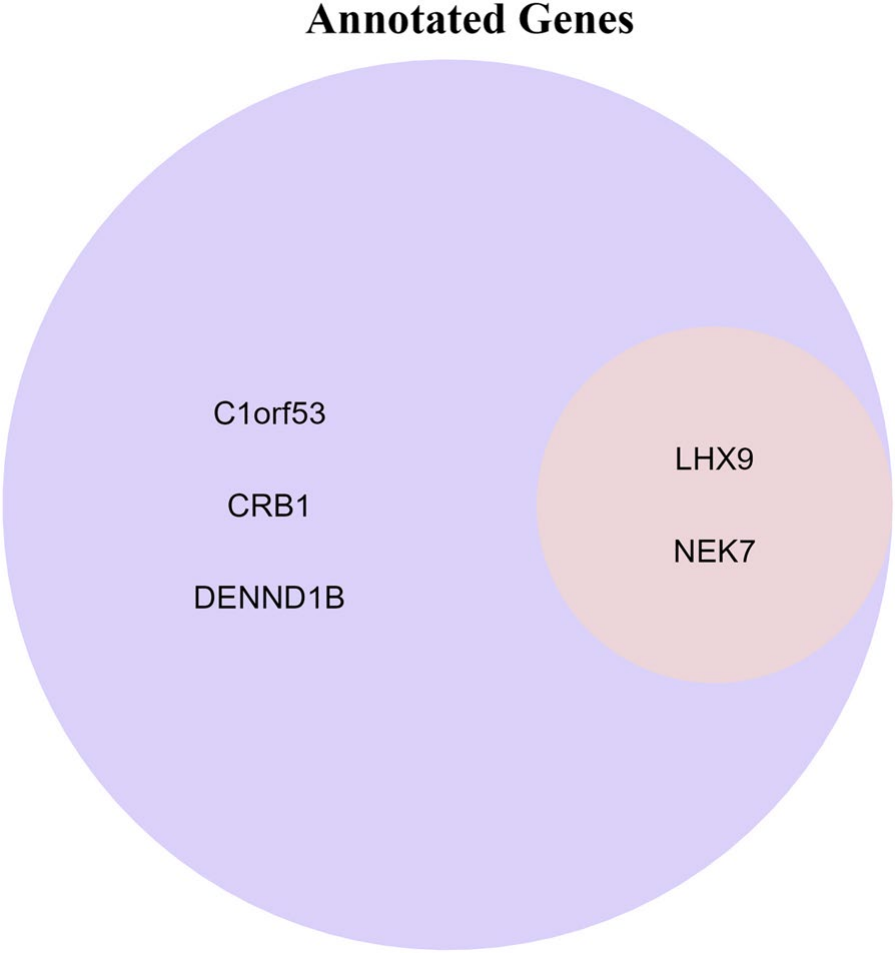
Mapped genes by FUMA and GWAS Catalog. Genes within the purple circle represent annotations derived from FUMA, while those within the light pink circle correspond to annotation information obtained from GWAS Catalog

As mentioned above, we have identified certain immune cells and innate SNPs as potential therapeutic targets, but the specific mechanism behind requires further investigation. Currently, it is widely accepted that single nucleotide polymorphisms are extensively involved in gene expression and modification through various mechanisms, exerting significant impacts on diverse physiological and pathological processes. To identify the regulated gene(s), we employed FUMA and GWAS Catalog to retrieve the target(s) most closely associated with SNPs. Totally, FUMA identified five potential regulated genes, including C1orf53, CRB1, DENND1B, LHX9 and NEK7. Meanwhile, information obtained from the GWAS catalog indicates the association with NIMA related kinase 7 (NEK7) and LIM homeobox 9 (LHX9) as a regulatory region variant (Fig. 3), reported by various studies before [27–29]. Collectively, this evidence suggests that rs17583875 may potentially play a role in colorectal cancer by regulating the expression of NEK7 and LHX9. To explore the plausibility of these gene as carcinogenic factors, we conducted an extensive literature retrieval and found several studies providing evidence supported the association between NEK7 and LHX9 with tumor initiation and progression.

Specifically, NEK7 has been suggested to be involved in cell cycle regulation as a member of NIMA-related kinases (NEK) family, and promoting tumor cell proliferation in cancer through relevant mechanisms. Elevated expression of NEK7 has also been observed in pancreatic ductal adenocarcinoma (PDAC) [30]and hepatocellular carcinoma (HCC) [31]. Downregulation of NEK7 has been shown to suppress pancreatic cancer liver metastasis and HCC progression [32]. In addition, LHX9 is demonstrated to orchestrate the reprogramming of glycolytic metabolism in gastric cancer [33] and glioma [34], while simultaneously exerting a pivotal influence on the intricate regulation of cell differentiation in neural cells [35].

**Fig. 4:**
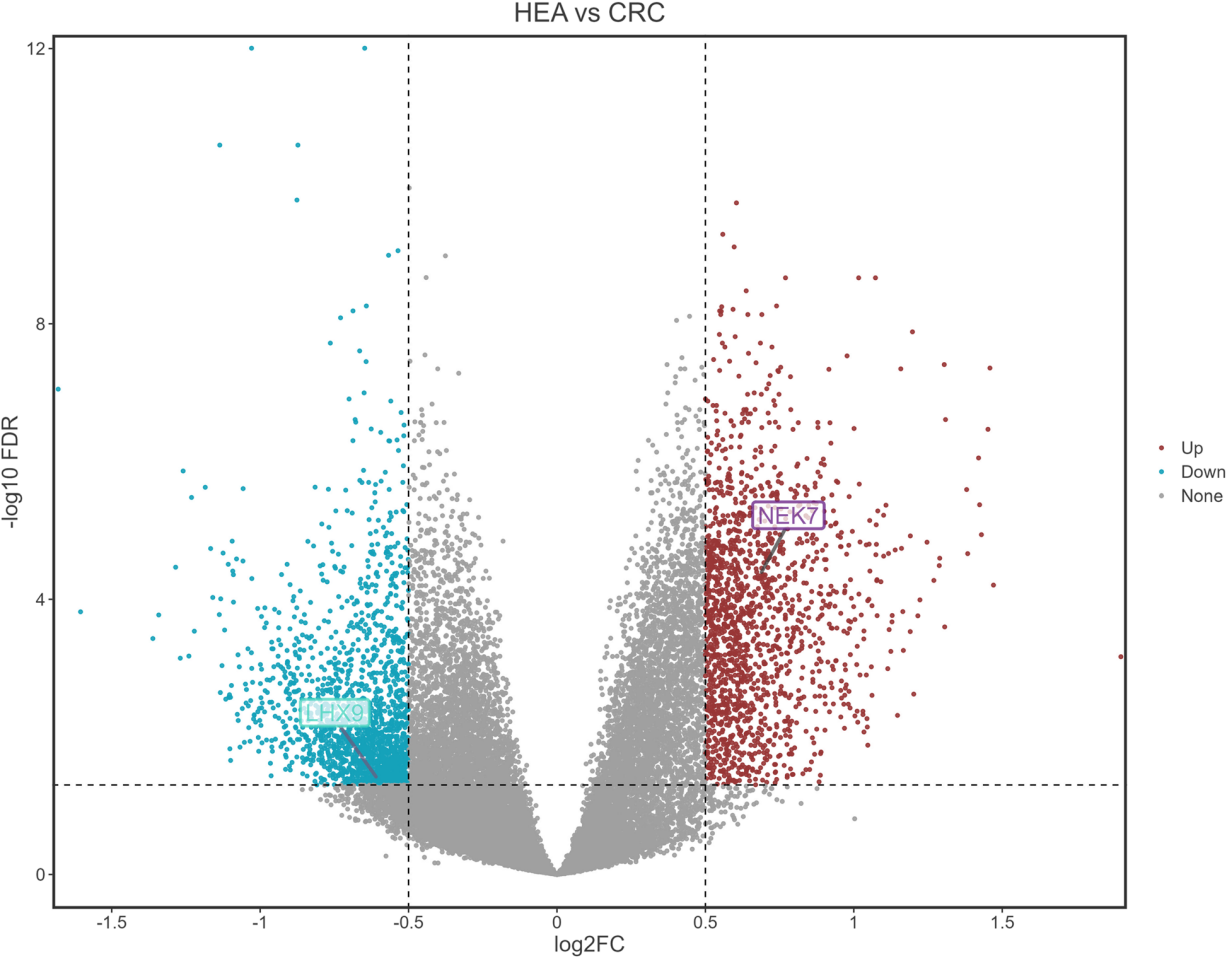
Volcano plot of differential analysis for GSE164191. Blue dots indicate genes with decreased expression levels, while red dots represent those with increased expression. Data compare healthy individuals to colorectal cancer patients

These findings demonstrate the important roles of NEK7 and LHX9 in driving tumor initiation and progression, and they are highly likely to be involved in the occurrence and progression of CRC as well. However, our current study is limited to the identification of these genes as potential effectors in immune cells, yet their presence and regulatory relationships with corresponding cell types remain uncertain. To address this gap, we then obtained one peripheral blood gene expression profile from the GEO database, including samples from CRC patients and healthy individuals. We then conducted a gene differential expression analysis and the results confirmed a significant increase in NEK7 expression and a decrease in LXH9 among CRC patients ( Fig. 4). To investigate the expression correlation, we proceeded with xCell analysis on three additional datasets and integrated with corresponding gene expression profiles.

**Fig. 5:**
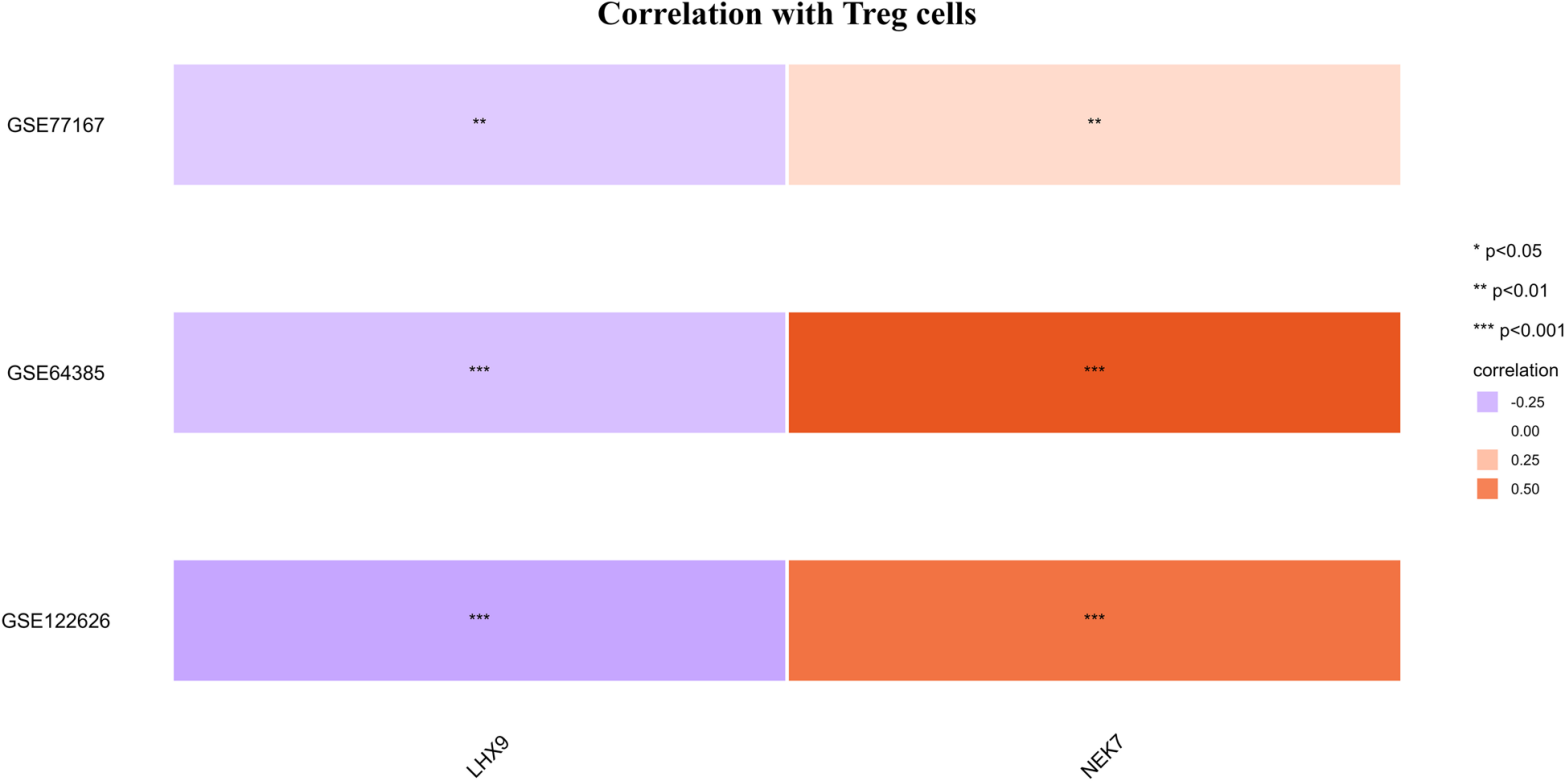
Correlation analysis graph of gene expression levels with Treg cells, derived from xCell analysis. Expression data are sourced from colorectal cancer patients within each dataset

Collectively, the result shows a positive correlation between transcriptional activity and Activated & resting Treg & CD4 + cells for NEK7, while a negative correlation is observed for LHX9 (Fig. 5), demonstrating a close functional association within them. This regulatory relationship is highly significant in CRC patients, and exhibits the same direction of regulation. By integrating the MR results, it can be inferred that the gene expression of NEK7 and LHX9 in Activated & resting Treg & CD4 + cell plays a crucial role in carcinogenesis and immune dysfunction. In conclusion, the analysis presented in this study supports our hypothesis regarding the involvement of peripheral Treg and DN cell functioning, specifically through gene regulation, in the progression of colorectal cancer.

### miRNA predicting

MicroRNAs (miRNA) contribute significantly to gene expression and silencing, while also participating in various aspects of cancer progression and diverse biological processes. At present, an increasing number of investigations are focusing their attention on the realm of miRNA-mediated cancer therapy, highlighting it as an exceptionally promising avenue for therapeutic advancement. To explore the druggability from this crucial perspective, we use the miRNet platform to identify miRNAs that interact with selected genes. The miRNet integrates 14 miRNA databases, encompassing over 380,000 miRNA-gene interactions of human. Giving that aforementioned analysis was based on circulating immune cells, the target miRNAs were specified in peripheral blood. To validate their presence and clinical relevance, we further employed the dbDEMC and OncomiR database to investigate their expression status in blood, as well as their biological associations from two distinct perspectives, including tumor stage and grade and survival outcome. The results retrieve a total of 11 miRNAs, with 11 for NEK7 and one for LHX9 (Supplementary Figure S3). Among those, has-mir-155-5p is associated with both LHX9 and NEK7. Data from dbDEMC indicates that almost all of these miRNAs show increased expression levels in the peripheral blood of colorectal cancer patients. However, out of the 11 miRNAs, four do not show any significant relevance to clinical parameters, suggesting a lack of convincing evidence. Other molecules are associated with at least one tumor pathological stage, and has-mir-21-5p is also significantly related to the survival of rectal adenocarcinoma patients with upregulated expression (Supplementary Table S7). In general, out of the 11 confirmed miRNAs, a total of seven miRNAs shows comparatively reliable associations with NEK7 and LHX9, while the remaining lack current support.

### Druggability of identified genes

To evaluate the draggability of selected genes, we searched several authoritative drug and molecule databases for current medicines targeting them. We also explored the interaction between genes and the targets of currently clinically used drugs for treating CRC using the STRING database. Previous study has shown that NEK9 has an activating effect on NEK7 [36], so molecules interacting with NEK9 may also have an impact on NEK7. Additionally, since NEK6 shares a high degree of similarity (87%) with NEK7 [37], we also documented relevant information about NEK6 as a more comprehensive supplement.

In total, 20 drugs and 11 bioactive molecules with updated information were documented **(**Supplementary Table S8-S9**)**. Drugs targeting NEK7 exhibit the highest abundance, while LHX9 only attracts one substance named otenzepad, an acetylcholine receptor antagonist designed to treat cardiovascular diseases, which surprisingly shows effectiveness for NEK7 as well. Most of them are currently undergoing investigation and experimentation, with only three having received approval as either nutrition supplements (Magnesium, Manganese) or hematological system drug (Fostamatinib) for treating rheumatoid arthritis. Five drugs and two molecules targeting NEK6 and NEK7 have already exhibited anti-tumor effects in investigations or clinical trials. Regarding miRNAs, currently available data only indicates four bioactive substances with the ability to regulate the miRNAs of NEK7, of which three have been authorized by the FDA, and the other one is a neurotoxin used to modeling Parkinson’s disease. These targeted miRNAs are all considered more reliable. However, no pertinent information on LHX9 was captured in this aspect, warranting further investigation and exploration. Due to the complexity of miRNA regulatory mechanisms, we did not further explore relevant information regarding NEK6 and NEK9 in this aspect. Moreover, the STRING database indicates that there’s no protein level interaction between commonly used drugs and identified genes.

## Discussion

We conducted a Two-sample MR to explore the causal relationship between circulating Treg and DN cells and CRC, providing relevant clues for the development of new treatment strategies. The results revealed a significant causal relationship between two immunocyte subtypes and colorectal cancer. Among them, Treg cells have potential tumor-promoting effects, and DN cells exhibit anti-tumor effects. Following the selection and utilization of genetic variants as analytical tools, we explored and demonstrated the underlying mechanisms of this association. Evidence suggesting that the expression of NEK7 and LHX9 may be potential reasons for the inverse correlation between Treg cells and CRC risks, whereas the factors for the anti-tumor effects of DN cells remains uncertain. We also attempted to identify the corresponding miRNAs for the aforementioned genes and conducted relevant analyses. Totally, 20 drugs and 11 molecules were identified to have potential effects in treating colorectal cancer through these immune cells.

The development and progression of colorectal cancer involves a variety of molecules and mechanisms, with immune cells playing a pivotal role. Treg cells have been identified as promising therapeutic targets for CRC, and DN T cells are also garnering increasing attention. In this study, we discovered a robust causal effect of circulating Treg and DN T cells on colorectal cancer through Mendelian randomization analysis.

To further explore potential biological mechanisms and clinical efficacy, multiple databases were utilized for in-depth analysis. Among all the Mendelian randomization results, only the SNP rs17583875 in the Treg cell group was significantly associated with the outcome in all three cohorts, demonstrating strong consistency. This suggests that, compared to other genetic factors, this SNP plays a more powerful and robust role in the pro-tumorigenic effects of peripheral Treg cells. Therefore, we retained only this genetic variant for subsequent in-depth analysis.

However, this does not deny the roles and importance of other genetic factors identified by MR in immune cells, and thus additional related studies are still necessary.

After integrating the analysis results from FUMA and the GWAS Catalog, NEK7 and LHX9 are considered to be the mediatory platforms involved in biological activities, participating in the pro-tumorigenic biological behavior of activated & resting CD4 regulatory T cell %CD4 T cells. FUMA provides potential genetic associations, while the GWAS Catalog compiles previous research, further enhancing the credibility of the associations.

To explore the therapeutic value of these genetic factors, we further conducted analyses on gene expression and the association with immune cells.

The results showed that NEK7 is significantly upregulated in CRC patients and exhibits a significant positive correlation with the expression of Treg cells. On one hand, this biologically substantiates the potential importance of NEK7 in colorectal cancer; on the other hand, it also suggests that Treg cells and NEK7 may influence each other—modulating Treg cells or NEK7 could have a significant impact on the role the other plays in tumor progression. This could potentially promote tumor development or exert an inhibitory effect.

Treg cells are well-known for their potent immunosuppressive effects, and this have established their intricate connection with cancer development: their abundant infiltration is significantly associated with poor prognosis in cancer patients [38], while the depletion of Treg cells can effectively counteract tumor progression [39]. Historically, most studies have primarily focused on the function of Treg cells within tumor and normal tissues, while comparatively fewer investigations have been conducted on the role of these cells in the peripheral blood. However, one study suggests that Treg cells in breast cancer may predominantly originate from the peripheral blood rather than conventional T cells, indicating their potential impact. Currently, numerous ongoing studies are dedicated to the depletion of Treg cells for anti-tumor purposes, while the identification of key targets that inhibit anti-tumor immune responses may potentially facilitate more effective and convenient advancements in cancer treatment research. Furthermore, apart from being used as a standalone treatment or in combination with other drugs for anti-tumor therapy, this targeted approach towards Treg cells can also serve as an adjunct to Treg cell-related cell therapy, enhancing treatment efficacy and preventing drug resistance. However, further extensive research is needed to analyze and validate the specific effects and certain molecular markers expressed by this particular subtype of Treg cells. NEK7, a member of NIMA (Never In Mitosis A)-related kinase family, has been reported to play a role in cell cycle regulation. It is also involved not only in normal cell proliferation but also in tumorigenesis and metastasis [30, 31]. One previous study shows a significantly increased expression level of NEK7 in colorectal cancer [40], and our study further proved the causal relationship between NEK7 and CRC. Mechanistically, NEK7 gene is involved in the formation of the mitotic spindle, and its depletion leads to fragile spindle formation, undoubtedly increasing genomic instability [41]. In this study, NEK7 presents significant association with immune cell mentioned above. However, similar to previous studies, we observed a significant increase in the expression of NEK7 in the peripheral blood of CRC patients, suggesting the functioning of other relevant mechanisms. Equally intriguing, a wealth of research indicates that NEK7 is also a major interacting partner of NLRP3, which has activation effects in both inflammasome activation and cell pyroptosis [42, 43]. NLRP3 can also be activated when mitochondrial dysfunction occurs [44]. However, there is currently a lack of research directly linking Treg cells with NEK7 for analysis. Given the crucial role of NLRP3 in the pathogenesis of IBD and the fact that intestinal inflammation is a major risk factor for CRC, with NLRP3 being a key inflammatory mediator directly involved in the pathological process of CRC [45]; in addition, as mentioned earlier, there are several close associations between NEK7 and the activation of NLRP3, along with our research on the causal relationship between NEK7 and CRC, it is therefore necessary to investigate the physiological and pathological role of NEK7 within circulating Treg cells in colorectal cancer.

However, these genetics-based studies only suggest the potential for molecular interactions, and there is still a gap to bridge before confirming their actual functional roles. We hope that our research can provide meaningful perspectives and insights for the diagnosis and treatment of colorectal cancer, paving the way for further investigation into its molecular mechanisms.

Therefore, to further explore these potential associations and aid in the diagnosis and treatment of colorectal cancer, extensive basic experiments and functional validations are required to ascertain the precise regulatory relationships between them.

Targeted drug therapies aimed at tumor cells or associated immune cells represent one of the most potent modalities in anti-tumor treatments, demonstrating remarkable efficacy in numerous cancers [46–50].Additionally, therapies related to miRNAs, which are closely associated with cell proliferation, differentiation, oncogenesis, and even the type and malignancy of tumors, are gradually coming into focus and hold broad application prospects [51, 52].Some molecular mimics or antagonists targeting miRNAs for cancer treatment have entered Phase I and Phase II clinical trial stages [53].Some studies have also found that circulating miRNAs play a significant role, not only possessing anti-tumor efficacy but also serving as a means for early screening, diagnosis, and monitoring of patient response to treatment [54]. Therefore, it is necessary to further explore the possibility of targeting genes through drugs and miRNA molecules.

However, it is important to note that our current research is limited to genetic association studies, and explorations and reports on related drugs should be more indicative in nature.

Having identified genes that may interact with pathogenic genetic factors, our initial hypothesis was that drugs could target the downstream transcriptional products of these genes, thereby exerting a series of beneficial effects. However, this approach is somewhat simplistic, and its efficacy and feasibility remain to be explored. Therefore, it is imperative to conduct further experiments and clinical studies to elucidate their potential value.

As previously mentioned, due to structural similarity or activating effects, we have included both NEK6 and NEK9 in our analysis. Across all drug databases, we identified a total of 20 different drugs, with 6, 16, and 5 targeting NEK6, NEK7, and NEK9, respectively. In terms of small molecule drugs, we found 1, 2, and 4 targeted substances for each, respectively.

Among all identified drugs, a noteworthy point is that among all documented drugs, Licochalcone B is capable of specifically inhibiting the NLRP3 inflammasome by interfering NEK7-NLRP3 interaction [55], making it a clearer candidate.

In terms of miRNA, we predicted three NEK7-related miRNA molecules and documented the associated drugs. We found that these miRNA molecules have been confirmed by research to be associated with CRC. Among them, mir-126-3p is associated with the gender of colorectal cancer patients [56] and the treatment toxicity of Regorafenib [57]. Mir-181a-5p can promote the transformation of fibroblasts into cancer-associated fibroblasts that are pro-angiogenic through the miR-181a-5p/RECK axis [58], and it can facilitate the metastasis of CRC to the liver via extracellular vesicles [59]. Mir-19b-3p can act as a biomarker to differentiate between healthy individuals and CRC patients, holding potential for the early diagnosis of CRC [60].

However, for various reasons, these studies have not linked miRNAs with their associated gene NEK7. Additionally, we have documented some small molecules targeting these miRNAs. By reporting which drugs and small molecules have the potential to regulate NEK7 and related genes within Treg cells, we aim to contribute to advancing the treatment of colorectal cancer. Through these analyses, we also hope to provide more valuable and meaningful references for drug therapies related to Treg and NEK7.

Compared to NEK7, investigations on LHX9 are relatively limited. Existing studies postulate its role in regulating glycolytic metabolism to get involved in the development of cancer, whereas the expression level is inconsistent in different tumors [34]. Mechanically speaking, in glioma, LHX9 was found to negatively interact with phosphoglycerate kinase 1 (PGK1), which dominates in the aerobic glycolysis pathway; while in gastric cancer, it mainly activates pyruvate kinase M2 (PKM2) to promote tumor progression. Both enzymes are observed to be highly upregulated in various cancers and may have an interactive relationship [61]. Additionally, studies found that LHX9 is often silenced by hypermethylation in follicular lymphoma [62] and child glioma [63]. In summary, LHX9 is of great importance in the regulation of biochemical pathways.

Similar to NEK7, we also attempted to predict miRNAs corresponding to LHX9 and conducted a comprehensive drug search. However, we did not identify any related miRNAs, and only found one potential drug that is currently in clinical trials for the treatment of cardiovascular diseases.

However, as previously mentioned, all current conclusions are based on further analysis of genetic associations found through Mendelian randomization and summaries of past research, lacking robust evidence such as foundational experiments.

Therefore, we anticipate that additional experiments will substantiate the aforementioned relationships, including the regulatory dynamics between Treg cells and NEK7, as well as the interactions between pharmaceuticals, miRNA molecules, and NEK6, NEK7, and NEK9.

Double negative T cells, belonging to a rare group of mature T cells in the peripheral blood, express either TCRαβ or TCRγδ while lacking both CD4 and CD8 molecules. Depending on the specific environment, DN cells can exhibits different functions: as T helper (TH) cells, DN cells can secrete cytokines like interleukin(IL)-17 and interferon-γ (IFN-γ) that may conduce to their protective effects against non-small-cell lung cancer (NSCLC) [14] and acute myeloid leukemia (AML) [64]; as cytotoxic T lymphocytes, they also possesses anti-tumor functions in leukemia [65] and lung cancer [15]. Our findings also indicate that DN cells may play a beneficial role in combating colorectal cancer. This implies that investigating the use of DN cells for anti-tumor therapy is justified and warrants further exploration. At the molecular level, however, it is frustrating that the specific genetic variants within identified DN (CD4-CD8-) T cell % leukocyte haven’t been confirmed. This indicates that additional research is still required to delve into the underlying mechanisms behind the profound anti-tumor effects exerted by DN cells. Overall, these immune cells and related genes play a significant role in various aspects of tumor biological activity. Investigating and exploring the impact of the immune system from the perspective of circulating immune cells, as well as conducting research on related biomarkers and therapeutic possibilities, is highly meaningful. As previously mentioned, this not only has the potential to empower pharmacological anti-tumor treatments but also holds promise in assisting with promising robust therapies such as CAR-Treg and adoptive immunotherapy, thereby advancing the development of anti-tumor clinical treatments and improving patient survival rates.

Due to ethnic differences, Treg and DNT cells may play roles that are not entirely identical in CRC patients from different regions. For instance, there may be lineage-specific variations in molecular expression levels and drug resistance. Concurrently, although many studies have demonstrated that these two types of immune cells generally exert a potent immunosuppressive effect, there are numerous factors that influence gene expression. The association between NEK7, LHX9, and Treg cells may be subject to a wide range of influences, thereby affecting their roles and status. Therefore, whether the conclusions of this study are applicable to other ethnic groups, such as Asians, requires further research and validation.

One notable strength of this study is the implementation of randomly assigned genetic variations to estimate the causal effects of immune cells on colorectal cancer. The Mendelian randomization analysis, along with Steiger’s test, effectively addresses confounding and reverse causality biases, thereby enhancing the credibility of the results. Furthermore, the utilization of large-scale data in MR and relevant association analyses enhances our ability to detect causal associations. Another significant advantage is the investigation in peripheral immune cell and the exploration of treatable targets in both drug and small molecule domains, which expands our horizons in cancer treatment. This approach opens up new possibilities for developing therapeutic interventions in the field of cancer research.

Some limitations in our study worth noting. Despite implementing sensitivity tests and corrections, completely eliminating potential horizontal pleiotropy remains challenging. However, employment of large-scale data may help mitigate the impact of pleiotropy. Additionally, our study only included a subset of immune cell types, limiting the comprehensive analysis of the causal relationship between other circulating immune cells and CRC, and potentially missing other effective traits. Simultaneously, our research and conclusions are currently limited to association studies based on genetics, and await further substantiation through additional experimentation. Moreover, our analysis includes only the European population; whether these findings are applicable to other populations remains to be explored. Finally, there is currently limited research on these specific genes within immune cells, leaving uncertainty regarding their potential as effective therapeutic interventions and the stage of gene expression at which intervention can be implemented.

## Conclusions

In summary, this study has revealed many associations between peripheral blood immune cells and colorectal cancer through the integration of genetic and other methods. Following a thorough analysis and retrieval, it has been determined that NEK7 and LHX9 within Activated & resting Treg %CD4 + cell, along with DN (CD4-CD8-) %leukocyte, hold promising potential as therapeutic targets for drug treatment of colorectal cancer. Further research and validation are required to explore how targeting specific cells can effectively modulate the expression of target genes, thereby preventing and treating tumor.

### Supplementary Information

Supplementary Material 1.

Supplementary Material 2.

## Data Availability

GWAS data used in this study can be downloaded from GWAS Catalog (https://www.ebi.ac.uk/gwas/). Gene expression data for healthy individuals and colorectal cancer patients were downloaded from the GEO database (https://www.ncbi.nlm.nih.gov/geo/).

## Abbreviations

CRC: Colorectal cancer
DN: Double negative
FUMA: Functional mapping and annotation
GEO: Gene expression omnibus
GWAS: Genome wide association study
IVW: Inverse-variance weighted
LD: Linkage disequilibrium
LHX: LIM homeobox
MR: Mendelian randomization
miRNA: MicroRNA
NLRP3: NLR family pyrin domain containing
NEK: NIMA related kinase
OR: Odds ratio
PGK: Phosphoglycerate kinase
PKM: Pyruvate kinase M
SNP: Single-nucleotide polymorphism
Treg: T regulatory
