# Supplementary material for: Genetically evaluating the causal role of peripheral immune cells in colorectal cancer: a two-sample Mendelian randomization study
Source: BMC Cancer. 2024 Jun 21;24:753. doi: 10.1186/s12885-024-12515-z (PMC11191266; doi:10.1186/s12885-024-12515-z)
Supplement: Supplementary file 1 — Supplementary Material 1. [file 12885_2024_12515_MOESM1_ESM.docx]

**SUPPLEMENTARY MATERIAL**

**Supplementary Figure 1.** Forest plots of the Mendelian randomization (MR) leave-one-out sensitivity analysis.

**Supplementary Figure 2.** Scatter plots of the Mendelian randomization (MR) main result based on IVW.

**Supplementary Figure 3.** Circle chart of identified miRNAs

**Supplementary Figure 1.** Forest plots of the Mendelian randomization (MR) leave-one-out sensitivity analysis.

Activated & resting CD4 regulatory T cell%CD4 T cell


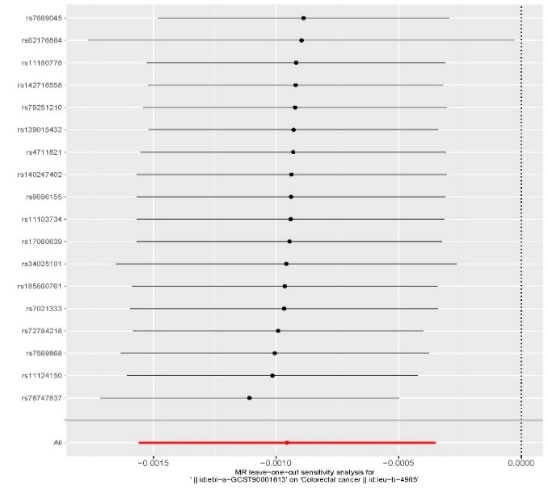


DN (CD4-CD8-) T cell % leukocyte


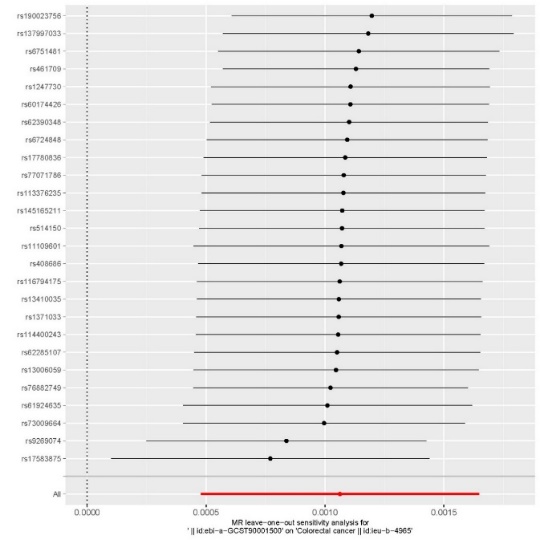


(1) UKB A group

**
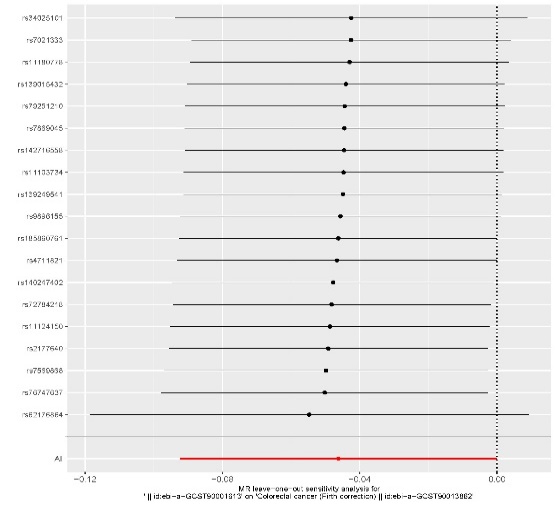

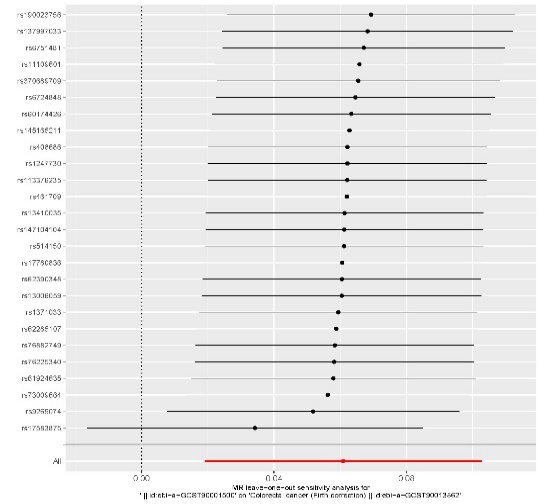
**

(2) UKB B group

**
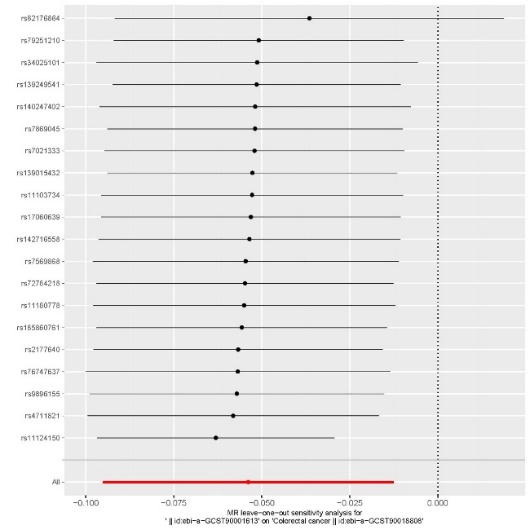
**


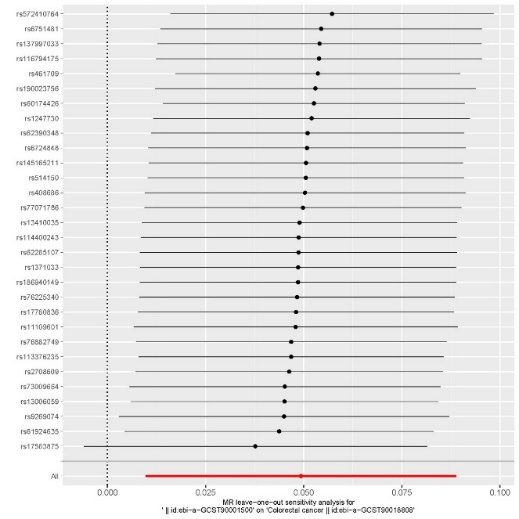


Figure S1 Leave-one-out analysis for the association of immune cells and CRC. These figures show the results of the leave-one-out analysis for all significant causal relationships identified in the primary analysis. The black dots represent the estimated causal association between a specific exposure and the target mental disorder when each SNP is removed in turn. The red dots represent the overall causal estimate using the random-effects inverse variance weighted method. The horizontal lines indicate the 95% confidence intervals. The results indicates that all SNPs identified in the study are qualified and steady.

(3) Meta-C group

**Supplementary Figure 2.** Scatter plots of the Mendelian randomization (MR) main result based on IVW.

DN (CD4-CD8-) T cell % leukocyte

Activated & resting CD4 regulatory T cell %CD4 T cell


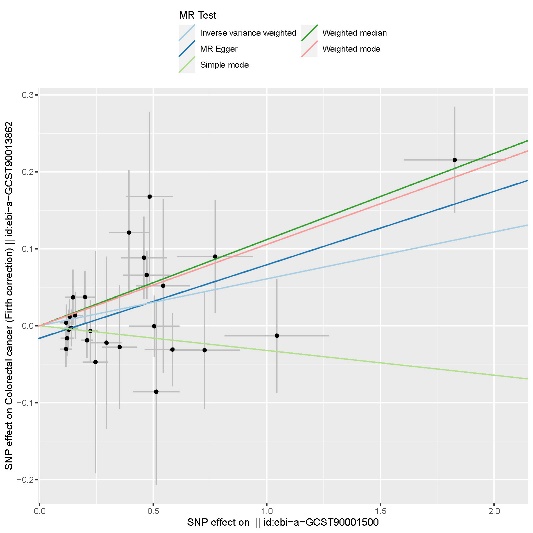

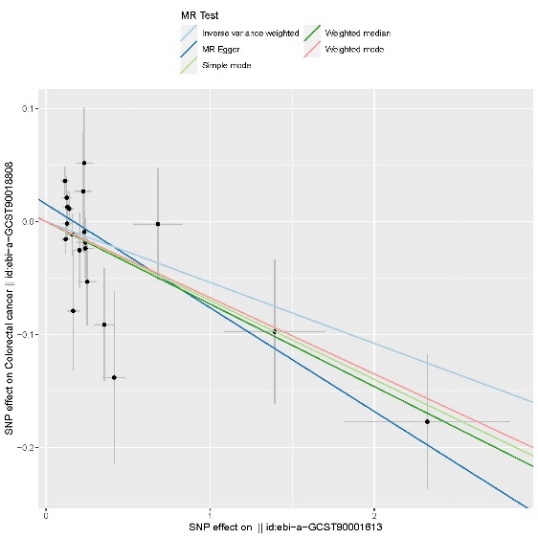
**
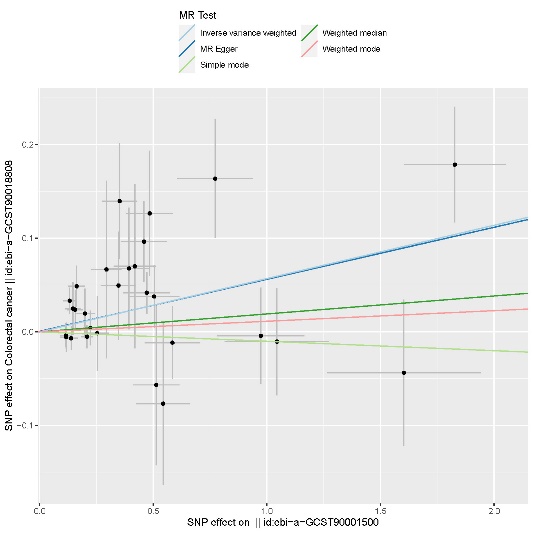
**
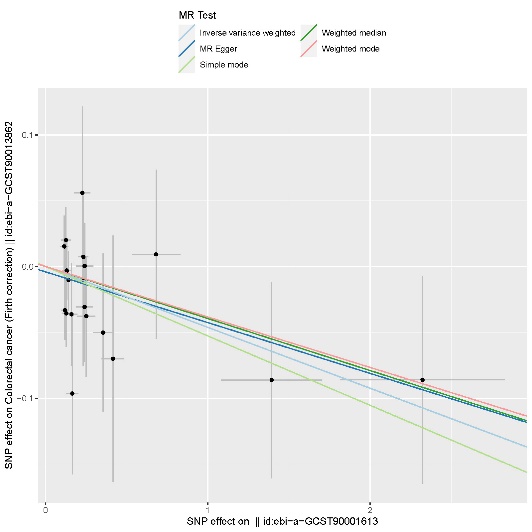
**
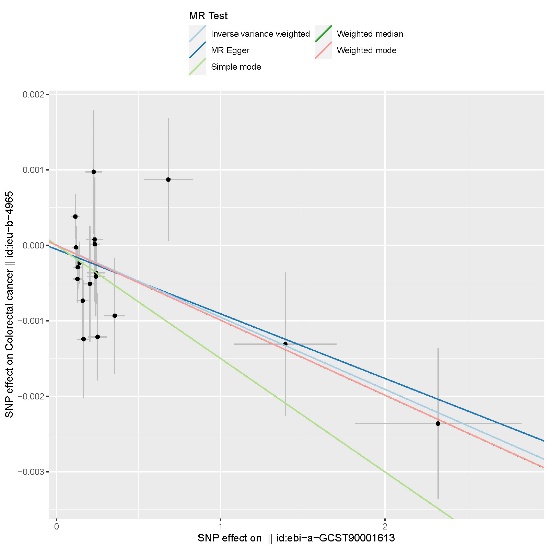
**
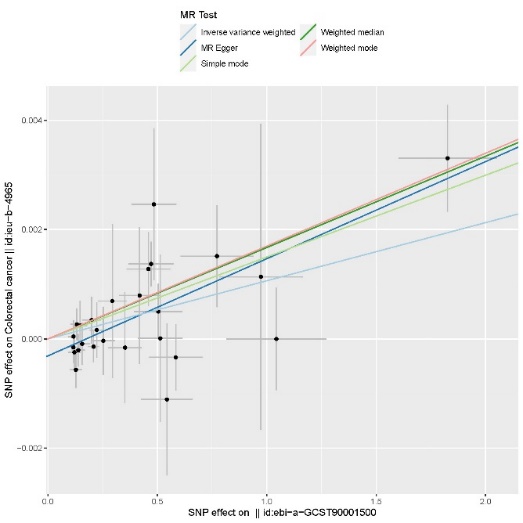


Figure S2. Causal effects of immune cells on CRC.

IVW, inverse variance weighted. The lines denote effect sizes. The scatter plots indicate that Activated & resting CD4 regulatory T cell %CD4 T cell promote the onset of colorectal cancer, while DN (CD4-CD8-) T cell % leukocyte have a protective role. Furthermore, they demonstrate that the direction of these effects is consistent across different groups.

(5) Meta C group

(2) UKB B group

(1) UKB A group

**Supplementary Figure** **3.** Circle chart of identified miRNAs

**
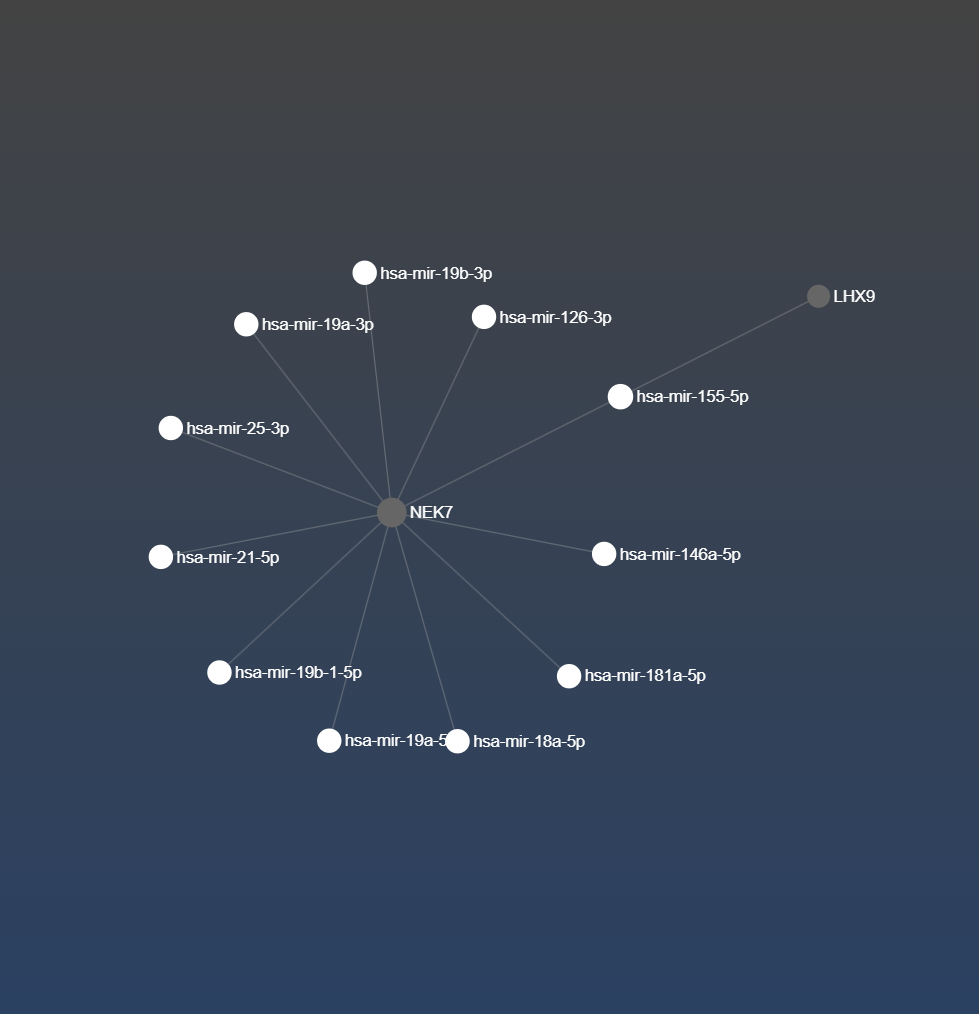
**
